# Atypical resource allocation may contribute to many aspects of autism

**DOI:** 10.3389/fnint.2013.00082

**Published:** 2013-12-26

**Authors:** Emily J. Goldknopf

**Affiliations:** Zaidel Lab, Department of Psychology, University of California Los AngelesLos Angeles, CA, USA

**Keywords:** autism, attention, resources, perception, cognition, action, language

## Abstract

Based on a review of the literature and on reports by people with autism, this paper suggests that atypical resource allocation is a factor that contributes to many aspects of autism spectrum conditions, including difficulties with language and social cognition, atypical sensory and attentional experiences, executive and motor challenges, and perceptual and conceptual strengths and weaknesses. Drawing upon resource theoretical approaches that suggest that perception, cognition, and action draw upon multiple pools of resources, the approach hypothesizes that compared with resources in typical cognition, resources in autism are narrowed or reduced, especially in people with strong sensory symptoms. In narrowed attention, resources are restricted to smaller areas and to fewer modalities, stages of processing, and cognitive processes than in typical cognition; narrowed resources may be more intense than in typical cognition. In reduced attentional capacity, overall resources are reduced; resources may be restricted to fewer modalities, stages of processing, and cognitive processes than in typical cognition, or the amount of resources allocated to each area or process may be reduced. Possible neural bases of the hypothesized atypical resource allocation, relations to other approaches, limitations, and tests of the hypotheses are discussed.

## INTRODUCTION

It was as if either my ears worked or my voice did but not at the same time. When I spoke, I heard noise but was deaf to most of the meaning I was making. I had to take it on trust that I was making meaning at all⋯. My brain was like a department store where the people running different departments were working alternate shifts. When one came to work, the others went to sleep⋯.

Williams (1994, pp. 95–96)

This account by Donna Williams, an autistic author, suggests a resource theory of autism, in which the processing of perception, action, and meaning is affected by limited neural resources. Of course, for autistic and neurotypical people alike, our theories of how our brains work may be wrong – we have access to our experience but not to the neural or psychological underpinnings of that experience. And given the great heterogeneity of people with autism, what is true of one autistic person’s brain may not be true of another’s. But what if her metaphor is correct? Can resource theoretical approaches contribute to our understanding of autism? This paper will develop one such approach, proposing that atypical resource allocation, which may be present to a greater or lesser extent in different people with autism, can be seen as a factor that ties together seemingly disparate symptoms and aspects of autism^1^.

Whereas many approaches to autism are centered on the three symptom areas in the DSM-IV-TR (American Psychiatric Association, 2000) diagnostic criteria – qualitative impairment in social interaction, qualitative impairment in communication and imaginative activity, and a restricted repertoire of interests and activities – a second group of aspects of autism has been noted clinically and experimentally. These include autistic people’s atypical sensory and attentional responses (e.g., Ornitz and Ritvo, 1968), movement issues (e.g., Damasio and Maurer, 1978), and unusual pattern of perceptual and conceptual strengths and weaknesses (e.g., Frith and Happé, 1994; Plaisted et al., 1998a). A number of approaches have focused on these other aspects of autism (e.g., Ornitz, 1989; Minshew and Goldstein, 1998; Plaisted, 2001; Murray et al., 2005; Happé and Frith, 2006; Mottron et al., 2006; Bonneh et al., 2008; Donnellan et al., 2013).

Building on this previous work, the present approach emphasizes these sensory, attentional, and perceptual/conceptual aspects of autism spectrum conditions (ASCs) while contributing to an explanation of the more classic criterial symptoms of autism, such as difficulties with language, social cognition, executive function, and action^2^. The approach draws upon resource theories of typical cognition that suggest that perception, cognition, and action draw upon a common resource or multiple pools of resources (e.g., Kahneman, 1973; Navon and Gopher, 1979) and especially upon Wickens’s multiple-resource approach to typical cognition Wickens (1980, 1984, 2002, 2008); it hypothesizes that compared with resources in typical cognition, resources in autism (especially in people with strong sensory symptoms) are (a) narrowed or (b) reduced^3^. In narrowed attention, resources are directed to smaller or fewer cortical areas, or to fewer cognitive stages or functions, than is typical. Attentional narrowing can occur within a sensory modality, between modalities, or within the larger canvas of cognitive functions and stages of processing. In some modalities, resources can be literally narrowed: in vision, to a smaller retinotopic or spatiotopic area; in somatic senses, to a smaller part of the body. This narrowed attention could be of typical intensity, or could be atypically intense, as if a typical amount of resources was being deployed to a smaller area. Resources can also be narrowed to one modality or cognitive process, or to fewer stages of processing. In possibility (b), which will be considered more briefly, overall resources are reduced. This may restrict resources to smaller areas, fewer modalities, or fewer processes or stages than is typical, or it may simply reduce the amount of resources allocated to each of these.

### SUMMARY OF HYPOTHESES

**(A) Narrowed Attention**

Resources are restricted to smaller areas and to fewer modalities, stages of processing, and cognitive processes than in typical cognition. Narrowed resources may be more intense than in typical cognition.

**(B) Reduced Attentional Capacity**

Overall resources are reduced. This may restrict resources to fewer modalities, stages of processing, and cognitive processes than in typical cognition, or it may reduce the amount of resources allocated to each area.

The approach does not suggest that conscious attention is needed for all stages of processing, but rather that resources underlying both attention and certain other aspects of processing in typical development are allocated atypically in autism. The approach also does not presume to suggest that atypical resource allocation is the only or even the primary factor in autism. Given the great heterogeneity of people with autism, there is a growing consensus that autism is multi-factorial, involving multiple genes (e.g., Abrahams and Geschwind, 2008) as well as possible epigenetic and environmental influences. Atypical resource allocation is most likely to be a factor in autistic people with strong sensory symptoms (hypo- and hypersensitivity).

In this paper, after briefly reviewing work on resource theory and attention, I will describe the current approach and discuss how it might address various symptoms and aspects of autism. I will then touch upon possible neural underpinnings of the approach, possible tests of the approach, limitations, and future directions.

## RESOURCES AND ATTENTION

### RESOURCE-THEORETICAL APPROACHES

A number of theories have attempted to explain perception, cognition, and action in terms of a pool or pools of resources; some such theories define the resource involved as attention. An example is Kahneman’s (1973) theory, which hypothesized that in addition to structural constraints, there is a general attentional upper limit on people’s ability to do mental work, including aspects of perceptual processing, the planning of action, and cognition; a variety of factors affect this capacity at any given moment. Subsequent experimental work supported the view that performance depends on multiple pools of resources (e.g., Navon and Gopher, 1979; Wickens, 1980), as will be discussed below. Much research in this area depends on comparing single-task and dual-task performance and in examining the amount of interference between tasks of different types, degrees of difficulty, and degrees of priority (Navon and Gopher, 1979).

Resource theories have received much criticism, including some from their own earlier proponents. It is hard to show that an effect arises from capacity limitations rather than from other causes. For example, when people do two tasks at once, each task may create *cross-talk* – outputs and side effects that interfere with the other task (Navon, 1984). In addition, people may switch their attention back and forth between multiple tasks rather than truly doing them simultaneously (Pashler and Johnston, 1998).

Despite these criticisms, work on resources has continued, especially by those concerned with ergonomics/human factors. Based on a meta-analysis of single- and dual-task experiments, Wickens (1980, 1984, 2002, 2008) has developed a resource theoretical model in which intersecting pools of resources are divided on three dimensions, each associated with a broad area of the brain: stages of processing (perceptual/cognitive vs. action, associated with processing posterior to or anterior to the central sulcus, respectively), codes (verbal vs. non-verbal, associated with the left and right hemispheres, respectively)^4^, and modalities (auditory vs. visual, associated with auditory and visual processing areas). In the most recent “3-D + 1” version, the three dimensions are supplemented by a distinction between visual channels (focal vs. ambient vision, associated with ventral and dorsal visual paths, respectively). Other multiple-resource approaches have focused on the cerebral hemispheres as independent pools of resources (e.g., Friedman and Polson, 1981), or, in a finer-grained analysis based on both subjective reports and behavioral studies, posit more numerous pools of resources (Boles et al., 2007). For present purposes, Wickens’s broad “3-D + 1” model will be used as a starting point in discussing resources.

Recent neuroimaging data appear to support the notion of resource limitations. There is increasing evidence that attention to one feature, spatial area, or modality is associated with a decrease in activation of cortical areas associated with other features, spatial areas, or modalities (e.g., Corbetta et al., 1990; Shomstein and Yantis, 2004). Shomstein and Yantis’s finding that selective attention to visual or auditory stimuli led to decreases in fMRI signal for the unattended modality may indicate that both modalities draw upon a shared perceptual resource pool, or might reflect cross-talk or inhibition between modalities.

### ATTENTION

The present approach also draws on notions of attention. As Pashler (1998) notes, the word *attention* may refer to a variety of phenomena, including selective attention (the gating, exclusionary process which enables some input to be processed further and some ignored) and attention conceived as a resource or capacity; both meanings are relevant to the current approach.

Selective attention can be considered in the context of Posner and colleagues’ influential approach, which distinguishes between three main attentional networks: alerting, orienting, and executive control (e.g., Posner and Petersen, 1990; Petersen and Posner, 2012). A simple behavioral test, the Attention Networks Test (ANT; Fan et al., 2002), is frequently used to test the efficiency and independence of the networks.

Work on the orienting network is most relevant here. In Petersen and Posner’s (2012) approach, the orienting network is associated with both a dorsal and a ventral system and with acetylcholine; it is responsible for prioritizing external stimuli by selecting a location or modality and is usually tested with cued attentional shifts (e.g., Posner, 1980)^5^. The dorsal system, involved in top-down visuospatial orienting, includes dorsal frontal areas, especially the frontal eye fields (FEFs), and dorsal parietal areas, especially the interparietal sulcus; the ventral system, involved in bottom-up reorienting, includes the right ventral frontal cortex and temporoparietal junction (Posner and Petersen, 1990; Corbetta and Shulman, 2002; Corbetta et al., 2008)^6^. Though the two systems work together (Corbetta and Shulman, 2002), the dorsal system is most relevant here. Similar but not identical dorsoparietal networks appear to be involved in controlling attention to stimuli in other modalities (Driver et al., 2004), in shifting attention between vision and audition (Shomstein and Yantis, 2004), and in attending to stimulus features such as color and motion (Corbetta and Shulman, 2002).

Also relevant is the question of what neurological process or state corresponds to attention, in the sense of the different amount or type of processing received by an attended-to stimulus or feature. Attention is associated with the modulation (usually the increase) of neuronal activation, with the result that attended-to input receives more processing while disattended input receives less (e.g., Corbetta, 1998; Reynolds, 2004). In work on the visual system, attention has been found to lead to greater neural responses for attended stimuli, to a decrease in suppression by competing stimuli, and to increases in baseline activity in the attended area (Kastner and Ungerleider, 2001).

The resource hypothesized in the present approach is conceived of as involving attention, or something closely underlying it, such as increased gain (Reynolds, 2004), a heightened signal-to-noise ratio, or increased baseline activation (Kastner and Ungerleider, 2001). It is hypothesized to underlie stages of processing of both external stimuli and internal representations.

I will now discuss the hypotheses in more detail and will examine how the atypical allocation of attention-like resources can contribute to many aspects of autism.

## HYPOTHESES

### RESOURCES IN TYPICAL COGNITION

To illustrate the allocation of resources in Wickens’s (1984, 2002, 2008) multiple resources approach, I will discuss two examples taken from typical development. (I sometimes distinguish between streams and stages of processing. *Streams* of processing operate largely in parallel; an example is the simultaneous processing of different sensory modalities. *Stages* of processing occur within a processing stream and are more sequential or cascading; an example is the movement in linguistic processing from phonetic information to meanings, and back again through feedback connections).

First, consider the example of drinking a cup of tea that one has been offered. One’s perception of the tea may include sight; sound (for instance, from the spoon); smell, touch, temperature, and proprioception. In Wickens’s (2008) view, sensory input from at least some of these modalities (vision and hearing) is partly separate but also draws upon a general perceptual pool. In the present approach, sensory input from these modalities is integrated and undergoes various stages of cognitive processing, involving schemas for the teacup, the tea, and the situation in which it has been offered; there is feedback from later stages to earlier ones. On the action side (which in Wickens’s (2008) view, draws upon a different pool of resources from perception/cognition), information flows from plans (for instance, to drink the tea) and motor schemas to motor acts (and back through sensory feedback).

Second, the comprehension and production of language also involves many stages of processing. People comprehending spoken language in face-to-face interaction must extract phonetic information, recognize words, and access their meanings; these processes (which in Wickens’s (2008) scheme draw upon auditory and verbal resource pools) may not be completely separate (e.g., Dahan and Magnuson, 2006). Hearers use semantic and syntactic information to combine the words into units, which are integrated into the ongoing discourse representation (e.g., Marslen-Wilson, 1989). In other streams of processing, hearers process prosody, recognize embodied aspects of the situation such as gestures or facial expressions, update representations of the interactional meaning of the utterance, and sometimes plan a reply. Many of these stages and streams of processing interact with each other. In spoken language comprehension, because new input rapidly arrives while previous input is processed, most stages of most processing streams probably operate simultaneously.

### THE HYPOTHESES IN AUTISM

Many symptoms of autism could be explained if we assume that the atypical allocation of resources (and more specifically, narrowed attention or reduced attentional capacity) affects streams and stages of the processing of stimuli, particularly meaningful stimuli. *Stimulus overselectivity*, in which children with autism have difficulty attending simultaneously to different modalities or different parts of the same modality (e.g., Lovaas et al., 1979), can be seen as an example of the effect of narrowed attention or reduced attentional capacity on parallel streams of perceptual input; see further discussion below.

For the more sequential stages of processing, I hypothesize that attentional narrowing or reduced attentional capacity makes it hard for people with autism to allocate resources to several stages of processing at once. In particular, I suggest that within the perceptual/conceptual resource pool, perceptual stages of processing, or other early stages, compete for resources with later or more conceptual stages of processing; in the action pool, plans compete with motor schemas. Although in Wickens’s (2008) scheme, perceptual/conceptual and action resources are separate pools, in autism, perception may compete with action.

The atypical allocation of resources to different stages of processing can be illustrated with Williams’s experiences of what she calls “meaning-blindness,” in which, particularly when under stress, she loses the meaning of visual and other stimuli. For example, referring to one of the many cups of tea which she was offered by a friendly couple, Williams describes herself as “sometimes not visually making meaning from this round white *chink-chink* thing with black *slop-slop* in it” (1994, p. 96); see the discussion above of perceiving a cup of tea. In the current approach, perceptual stages may receive an atypically large share of resources and conceptual stages may receive an atypically small share.

I will now examine how the hypothesized atypical resource allocation could contribute to a number of areas in autism.

## APPLICATION OF THE APPROACH TO ASPECTS OF AUTISM

### SENSORY ASPECTS

Children and adults with ASCs have long been noted to have atypical sensory responses and experiences, including sensory hypersensitivity, hyposensitivity, and a tendency to seek sensory stimulation (e.g., Ornitz and Ritvo, 1968); atypical sensory responses are also found in Asperger syndrome (e.g., Dunn et al., 2002). Though atypical sensory experiences are not specific to ASCs, studies based on parental and self-report have found more sensory symptoms in autism than in control groups (e.g., Rogers et al., 2003; Minshew and Hobson, 2008; see Ben-Sasson et al., 2009 for a meta-analysis), and sensory symptoms are now included in the DSM-V (American Psychiatric Association, 2013).

Sensory differences are reported in many first-person accounts by people with ASCs (see, e.g., Bogdashina, 2003; Donnellan et al., 2006; Robledo et al., 2012). In a book based on such accounts and on her experiences as the director of a day care center for autistic children, Bogdashina hypothesizes that the perceptual experience of people with ASCs fluctuates between hypersensitivity, hyposensitivity, and typical perception; this hypothesis is supported by a study (based on parental report) of children with autism that found that measures of sensory overreactivity and underreactivity were correlated in 43% of the sample (Liss et al., 2006).

According to Bogdashina (2003), other atypical phenomena reported in autism include fragmentary perception (in which a single modality is focused on or objects are seen in pieces), delayed perception (in which memorized strips of sensory input may be analyzed at a later time), synesthesia, and *sensory agnosia* (difficulty in interpreting the meaning of sensory input). Bogdashina (2003) and Williams (1992, 1994) describe a phenomenon called “overload,” in which, especially under conditions of stress and anxiety, sensory input appears to be amplified and sometimes snowballs into an overwhelming multisensory experience. This sometimes leads to what Williams (1992) calls “shutdown,” in which she feels nothing.

In the present approach, atypical resource allocation may contribute to sensory abnormalities such as sensory hyper- and hyposensitivity. Narrowed (but intense) attention may involve the atypical focusing of attentional resources on or within an early sensory processing area, leading to sensory hypersensitivity through such mechanisms as the firing of more neurons or increased gain control. This is consistent with findings that in hearing, stimulus intensity can be encoded through the number and frequency of neurons firing (Gulick et al., 1989), and that even covert attention can increase the response to an auditory stimulus at a location (Spence and Driver, 1994). Conversely, such an intense focusing of attention-like resources on one modality could decrease resources devoted to other modalities, resulting in sensory hyposensitivity or extinction-like processes (Bonneh et al., 2008), and helping explain stimulus overselectivity and other attentional narrowing in autism (discussed below). Fluctuations in the amount of resources devoted to a modality may result in the sense that the input itself is fluctuating (The opposite possibility, that atypical sensory processing in autism may affect the allocation of resources, will be considered in the neural underpinnings section below).

### ATTENTION

Some aspects of attention in autism, including orienting to stimuli, shifting attention, and the breadth of the attentional focus appear to be atypical in ASCs, though there have been some mixed results (Burack et al., 1997).

#### Shifting attention

Of the work on shifting attention in autism, work on spatial orienting – on shifting attention between spatial locations – and also on shifting attention between modalities is most relevant^7^. Studies of visuospatial orienting often distinguish between exogenous (automatic or reflexive) and endogenous (voluntary) orienting, as well as between orienting which is overt (using movements of sensors such as the eyes) and covert (using only attention; Burack et al., 1997). There is conflicting evidence about whether young children with autism are slower (Landry and Bryson, 2004) or as fast as or faster (Leekam et al., 2000) than controls to disengage overt attention from a central stimulus and attend to a peripheral stimulus. With respect to covert shifts of visual attention, individuals with ASCs (unlike age-matched controls) did not shift covert attention in response to valid cues at short cue-target intervals, while (like controls) they did shift attention at longer cue-target intervals (Wainwright-Sharp and Bryson, 1993). Slowed voluntary covert orienting in autism has been associated with cerebellar and parietal abnormalities (Townsend et al., 1996) and with diminished activation in fronto-cerebellar spatial attention networks (Haist et al., 2005). Some have suggested that problems with symbolically cued attentional shifts in autism may partly stem from difficulty in interpreting the cues (Burack et al., 1997; Leekam et al., 2000).

Using the ANT, Keehn et al. (2010) found that the orienting network was less efficient in children and adolescents with autism. Based on their findings and on a review of literature on the three attentional networks in autism, Keehn et al. (2013) suggest that impaired disengagement of attention may lead to atypical perceptual processing and to impairments in arousal regulation, attentional shifting, and joint attention, contributing to social-communicative impairments in ASCs.

Turning to shifting attention between modalities, in a study on task switching in children with autism and two mental age-matched control groups, Reed and McCarthy (2012) found that the autistic children performed worse than controls when switching between two visual tasks; they were especially impaired when switching between auditory and visual tasks. Noting that people with autism often have difficulty in switching between multiple cues and in shifting attention once engaged in a task, Reed and McCarthy (2012) point out that social communication often involves attentional shifts and cross-modal input. They conclude that their results indicate impaired cross-modal attention shifting in autism^8^ and that such impairments may contribute to social and communicative difficulties in autism.

Suggestions that difficulty in disengaging attention (Keehn et al., 2013) and in cross-modal switching (Reed and McCarthy, 2012) may contribute to social-communicative impairments in ASCs are reasonable. They are compatible with the hypothesized atypical resource allocation, which, as suggested below, may contribute to problems with shifting attention. Longitudinal studies, as well as correlations among these difficulties and with measures of social communication, may help clarify how each ability contributes to the development of social communication in typical development and autism.

#### Breadth of attention

The general picture in autism is one of attentional narrowing, though there has been some mixed evidence.

Early studies of autism found evidence for stimulus overselectivity, a tendency to respond to only part of a complex stimulus, both within and between modalities (e.g., Lovaas et al., 1979). Though not exclusive to autism, and associated with intellectual level (Schover and Newsom, 1976), stimulus overselectivity has been found to be greater in ASCs even when mental age is controlled for (e.g., Rincover and Ducharme, 1987; Leader et al., 2009). Stimulus overselectivity is often thought to arise from attentional narrowing during stimulus presentation, a view supported by findings that participants with intellectual disability look less at underselected parts of the stimulus (Dube et al., 2003). Another view, that stimulus overselectivity occurs at retrieval and is increased by an oversensitive “comparator” in autism, is supported by findings that when the overselected stimulus was extinguished, the underselected stimulus reemerged to control behavior; in autism, this was only found in participants without intellectual disability (Reed et al., 2009; Reed, 2011). In terms of the present approach, narrowed or reduced attentional resources, deployed to salient or highly reinforced aspects of stimuli, could contribute to stimulus overselectivity during both stimulus presentation and retrieval.

Electrophysiological and neuroimaging work on the breadth of attention in autism has had mixed results. In an event-related potential (ERP) study of covert visual attention in autistic participants with cerebellar abnormalities, Townsend and Courchesne (1994) found that whereas in controls, P1 components (taken to reflect attention-related processing enhancement) decreased steadily around a central focus, in five autistic participants with parietal abnormalities, these components showed a sudden drop-off around the central focus, whereas in three autistic participants without parietal abnormalities, the components showed an atypically broad pattern. In an fMRI study, participants were cued to covertly shift attention from one visual field to the other while also pointing in the direction of the shift (Belmonte and Yurgelun-Todd, 2003). In controls, fMRI signal from contralateral early visual processing areas switched back and forth along with the cued attentional shifts; in autistic participants, the signal was not modulated by the shifts. The authors concluded that in autism, activation in early visual processing areas is not modulated by attention but instead is atypically intense and broadened, with unattended stimuli possibly being suppressed at a later stage. While early sensory activation in autism may indeed turn out not to be modulated by attention, the autistic participants may also have had difficulty in shifting attention back and forth and may have strategically broadened their attention.

Two recent studies may shed light on the breadth of attention in autism. In one (Mann and Walker, 2003), the authors concluded that rather than having permanently narrowed attention, autistic people may have difficulty in broadening visual attention once they have narrowed it. In Bonneh et al.’s (2008) case study of a male adolescent with autism, when stimuli were presented simultaneously or in rapid succession, the perception of some stimuli interfered with the perception of others: auditory stimuli interfered with stimuli in other modalities, and color stimuli interfered with form stimuli. However, there were no signs of spatial extinction: perception of stimuli on one side of space did not interfere with perception of stimuli on the other side. Bonneh et al. suggest that these effects may reflect a non-spatial form of extinction; this hypothesis will be discussed more below.

First-person accounts describe the experience of narrowed attention in autism. Writing about her childhood, Williams reports that when she touched her leg, she typically could feel either her hand or her leg, but not both at once (Williams, 1994, p. 232). Tito Mukhopadhyay, a severely affected but literate boy with autism who was 14 when interviewed, describes difficulty in experiencing more than one modality at a time and in switching between modalities (Blakeslee, 2002). Mukhopadhyay says that when he was younger, he didn’t feel sensation in his body except when in the shower or hungry; he implies that he hand-flaps partly to regain a sense of his body (Blakeslee, 2002).

The recent *intense world syndrome* approach to autism addresses narrowed attention among other phenomena (Markram et al., 2007). In this approach, based on an animal model of autism in rats prenatally exposed to valproic acid, sensory hypersensitivity in autism is based on the hyper-reactivity of local neuronal circuits, and fragmentary perception is based on “*hyper-attention*,” which involves “hyper-focusing on fragment(s) of the sensory world with exaggerated and persistent attention” (Markram et al., 2007, p. 87). Markram et al. (2007) suggest that difficulty in shifting attention in autism may stem from difficulty in controlling these hyperactive microcircuits.

The present approach builds on these earlier approaches to narrowed attention in autism, suggesting that in addition to narrowed attention in the sensory and perceptual world, resources in people with autism (and especially in those with strong sensory symptoms) are narrowed to fewer stages of processing, affecting perception, cognition, and action.

#### Arousal

Atypical levels of arousal have long been suspected in autism; hypotheses have included chronic over-arousal (Hutt et al., 1964) and fluctuating arousal (Ornitz and Ritvo, 1968). Linking hypothesized intermittent over-arousal in autism with hypotheses that over-arousal leads to the restricted utilization of cues, Kinsbourne (1987) suggested that over-arousal in autistic children may lead to stimulus overselectivity, stereotypies, and sensory avoidance. Recently, there has been renewed interest in the role of arousal in autism (e.g., Toichi and Kamio, 2003; Anderson and Colombo, 2009). In relation to the present approach, questions include whether over-arousal could lead to the hypothesized narrowed attention, and whether such attentional narrowing could extend to different levels of processing.

#### Possible role of atypical resource allocation in attention in autism

Narrowed attention and reduced attentional capacity can contribute to difficulties with rapid voluntary shifts of spatial attention in autism in at least two ways. First, as others have noted, problems in interpreting symbolic cues may contribute to such difficulties; atypical resource allocation may affect the comprehension of symbols, as discussed in the language section below. Second, as Bonneh et al. (2008) note, it may be harder to inhibit an intense attentional focus to start an attentional shift. If, as Townsend and Courchesne (1994) suggest, an intense central focus of attention is surrounded by diminished peripheral attention in autism, it may also be harder to boost activation in those peripheral areas. Similarly, a narrowed (and possibly intense) focus on one modality could contribute to the slowed cross-modal attention shifts found by Reed and McCarthy (2012). Finally, the intense activation of early processing areas could lead to diminished activation of the areas that control and shift attention, including the frontal and parietal areas noted by Petersen and Posner (2012).

#### Does narrowed or broadened attention come first in autism?

In seeming contradiction to the present hypotheses are suggestions that people with ASCs may sometimes have broadened perceptual attention. Autistic people’s vulnerability to sensory overload as well as tendency towards synesthesia (Bogdashina, 2003) may reflect intense sensory activation that spreads between modalities. Bogdashina (2003) suggests that to avoid sensory overload, people with ASCs may tend to be aware of only one modality at a time, though processing without awareness may occur in other modalities. Belmonte and Yurgelun-Todd (2003) suggest that atypically broad and intense early processing of sensory input leads to suppression at later stages of processing. In terms of the present approach, these suggestions raise the questions: is narrowed attention a primary phenomenon in ASCs, or a response to intense or spreading sensory activation? At what point in development, and at what stages of processing, does such narrowing occur?

### PERCEPTUAL/CONCEPTUAL STRENGTHS AND WEAKNESSES

People with autism have unusual perceptual and conceptual strengths and weaknesses. Children with autism tend to do well on tasks that involve ignoring context, such as the embedded figures test, and tend to do poorly on tasks requiring the interpretation of stimuli in context, such as the disambiguation of homographs (Frith and Happé, 1994). This pattern has been described as Weak Central Coherence (WCC; a diminished drive to integrate information into higher-level contextualized representations), more recently conceptualized as a local processing bias (Happé and Frith, 2006).

Some perceptual abilities are enhanced in autism. People with autism were better than controls at a task involving discriminating patterns of small circles (Plaisted et al., 1998a). People with autism are generally faster than controls at visual search tasks, including tasks involving targets formed by conjunctions of features, possibly due to a greater ability to discriminate between stimuli (e.g., Plaisted et al., 1998b).

In contrast, studies have found that autistic people are less good than controls at detecting a variety of types of motion, including global motion; it has been suggested that this is due to problems with the dorsal visual pathway, which receives predominantly magnocellular input (e.g., Spencer et al., 2000). However, autistic participants were only impaired at detecting complex motion and not at detecting simple motion (Bertone et al., 2003); in a static task, they were better than controls at detecting simple sine gratings but worse at detecting more complex gratings (Bertone et al., 2005); Bertone et al. (2005) argue that it is not magnocellular processing or motion that is more difficult in autism, but rather, stimulus complexity.

Superior performance on simple perceptual tasks coupled with difficulties on more complex tasks is one of eight principles of autistic perception suggested by Mottron et al. (2006) as part of the Enhanced Perceptual Functioning (EPF) model^9^. Another principle is greater autonomy of perception from top-down influences (e.g., Soulières et al., 2007), which may help explain why people with autism tend to be less susceptible to visual illusions (e.g., Mitchell et al., 2010).

Some of the most striking strengths and weaknesses in autism are seen in autistic savants, who have extraordinary abilities in areas such as memorization, calculation, drawing, or music, but who may have intellectual deficits; diminished top-down influences have also been proposed as being involved (e.g., Snyder and Mitchell, 1999). Recently, Mottron et al. (2013) have suggested that savants have *veridical mapping* (VM), in which perceptual domains are mapped onto homologous perceptual or abstract domains, and that VM may also lead to phenomena such as hyperlexia, absolute pitch, and synesthesia in non-savant autistic people.

#### Role of atypical resource allocation in perceptual strengths and weaknesses

Atypical resource allocation can help explain this pattern of strengths and weaknesses. Narrowed attention in ASCs could lead to a tendency to focus on smaller areas within a modality and to ignore context, contributing to superior performance on tests such as the embedded figures test (Happé and Frith, 2006). In addition, within the perceptual/conceptual resource pool, the atypical allocation of resources to early processing stages may lead to less distraction from later stages of processing. The same patterns of resource allocation could lead to poor performance on tests that involve evaluating stimuli in context.

The allocation of additional attentional resources to early sensory processing areas can contribute to enhanced sensory and perceptual discrimination in autism (e.g., Plaisted et al., 1998a; Bonnel et al., 2010), perhaps by increasing gain control, signal-to-noise ratio, or baseline activation. Structural differences, such as altered lateral connectivity (Kéïta et al., 2011) or more numerous narrower minicolumns (Casanova et al., 2002) may also be involved. Autistic people’s difficulties with more complex stimuli (e.g., Minshew and Goldstein, 1998; Bertone et al., 2005) may stem from the effects of narrowed or reduced resources on the number of cortical areas or stages involved rather than from complexity *per se*.

Both the present approach and the EPF model are supported by an excellent recent meta-analysis of functional neuroimaging studies of visual processing of faces, objects, and words in autism (Samson et al., 2012). The meta-analysis, which focused on studies from which Activation Likelihood Estimation (ALE) maps could be computed, found that autistic participants generally had greater activation than controls in posterior regions (temporal, parietal, and occipital cortices), but less activation than controls in frontal areas. Samson et al. (2012) suggest that perceptual processing (and especially visual processing) may play a larger role in cognition in autism than in controls.

### EXECUTIVE FUNCTION AND MOVEMENT

#### Executive function

Executive functions, including planning, shifting mental set, generating alternative actions, and inhibition (Hill, 2004), are thought to be subserved by the frontal lobes. Problems with executive function have been proposed as a primary deficit in autism and as an alternative explanation for difficulties with theory of mind tasks (Ozonoff et al., 1991). While some studies of people with autism have found problems with aspects of executive function, such as shifting set (Ozonoff et al., 1991) and inhibiting responses (Hughes and Russell, 1993) other studies have found less evidence of executive problems or have linked them with developmental level rather than with autism *per se* (e.g., Griffith et al., 1999).

The hypothesized atypical resource allocation is especially compatible with Ozonoff’s (1995) account of executive function in autism. Drawing upon work on the role of the prefrontal cortex in holding representations on-line as a guide to action, Ozonoff (1995) suggests that what is common to the executive function tasks is an ability to “disengage from the immediate environment or external context and guide behavior instead by mental models or internal representations” (p. 201). Ozonoff (1995) hypothesizes that an inability to hold mental representations on-line may explain autistic people’s difficulties with theory of mind tasks, emotion perception, imitation, spatial reasoning, and pretend play.

Atypical resource allocation in autism can help explain difficulties in holding a mental representation online as a guide to action. It is often assumed that action has a hierarchical structure, involving a continuously updated overall plan as well as smaller goals and motor actions, and that motor acts involve perceptual and proprioceptive feedback. Given these assumptions, narrowed attention or reduced attentional capacity may make it difficult to simultaneously allocate resources to plans and action schemas and to monitor perceptual and proprioceptive feedback.

#### Movement

Movement differences and disturbances have long been noted in ASCs (e.g., Kanner, 1943; Damasio and Maurer, 1978). Movement differences in autism include apraxia, atypical postures, repetitive behaviors such as hand-flapping, and difficulty in starting or stopping movements (Donnellan et al., 2006). Donnellan et al. (2006) suggest that while such movement differences have often been regarded as volitional “autistic behaviors,” they can be more fruitfully seen as reflecting neurological differences, just as tics in Tourette Syndrome are seen as reflecting neurological differences. Donnellan et al.’s (2013) approach to autism centers on such sensory and movement differences, and broadens movement to include aspects of emotion and thought.

Whyatt and Craig (2013) found that people with autism have special difficulties with prospective movements such as catching a ball, in which the movement must connect with an external moving object; they suggest that this is due to problems with perception-action coupling and the spatiotemporal control of movement.

Torres and colleagues hypothesize that differences in stochastic signatures of spontaneous vs. goal directed movement form a source of kinesthetic/proprioceptive afferent feedback that helps children develop intentional movements (Brincker and Torres, 2013; Torres, 2013; Torres et al., 2013). In a case study of an autistic adolescent and controls learning a martial arts sequence, Torres (2013) found that goal-directed and spontaneous movements were stochastically distinguishable in the controls but not in the autistic participant, whose movements were also very similar to one another. In a later study of movement in ASD and TD participants over a wide range of ages, Torres et al. (2013) found that the ASD participants’ movements were more similar to those of young TD children, with a narrower bandwidth of speeds but also less predictable variability across trials. Torres et al. (2013) suggest that older ASD participants may compensate for their lack of kinesthetic learning by relying on other means such as visual feedback. Torres et al. (2013) suggest that noisy, unreliable movement in autism contributes to difficulties interpreting others’ movements and may contribute to a preference for sameness and to social impairments.

Atypical resource allocation can contribute to movement differences in autism in a variety of ways. Narrowed attention could lead to a lack of proprioceptive and tactile perception of one’s own body parts, probably making it harder to initiate movements. Prospective motions such as those studied by Whyatt and Craig (2013) should be even harder for people with narrowed attention because real-time awareness of one’s own body movements must be integrated with information about the object’s ongoing trajectory. In the Torres (2013) and Torres et al. (2013) studies, decreased real-time proprioceptive feedback could lead movements to be more ballistic and similar in speed as well as to a lack of kinesthetic learning and less predictable variability across movements.

While the present approach may contribute to an explanation of some of these differences, it cannot fully explain this rich area. For instance, whereas a lack of proprioceptive feedback might contribute to difficulty in initiating movements, another cause might involve reduced dopamine, as found in Parkinson’s disease.

### LANGUAGE AND COMMUNICATION

As reflected in the DSM-IV criteria, language is usually delayed and sometimes absent in autism, and non-verbal communication is also affected. Syntactic and phonological development in autism, though reflecting general language delay, are usually less affected than some other aspects of language, though some children with autism have severe phonological problems (Tager-Flusberg et al., 2005). While many researchers attribute language and communication impairments in autism to social deficits, autistic children’s early oral and manual-motor skills have been found to strongly correlate with their later speech fluency, implying that for at least some autistic people whose language is absent or delayed, motor issues may be involved (Gernsbacher et al., 2008). As discussed in the previous section, atypical resource allocation can contribute to these motor difficulties.

The present approach is especially relevant to three aspects of language and communication that tend to be strongly affected in ASCs: prosody, pragmatics, and semantics.

#### Prosody

Prosody is often impaired in both autism (Baltaxe et al., 1984; Tager-Flusberg et al., 2005) and Asperger’s syndrome (Klin and Volkmar, 1997). In terms of the present approach, problems with the production of prosody in autism may result from difficulty in hearing others’ meaning and prosody at the same time, making it harder to learn prosodic norms (Schreibman et al., 1986); from autistic people’s difficulty in speaking and listening to themselves at the same time, making it hard for them to monitor their own prosody (Bonneh et al., 2008); and possibly from motor issues.

#### Pragmatics

Pragmatic impairments in autism range from early difficulties with eye contact and joint attention (Mundy et al., 1993) to later difficulties with politeness, social register, and orienting to interlocutors’ interests and knowledge (Tager-Flusberg et al., 2005); despite relatively spared language abilities, people with Asperger’s tend to have pragmatic difficulties (Klin and Volkmar, 1997). Pragmatic impairments in autism and Asperger syndrome are usually attributed to social impairments in these conditions, but as discussed below, semantic factors and resource allocation may also play a role.

#### Semantics

There is mixed evidence about word use, reading, and semantic processing in autism. While autistic children without intellectual disability tend to do well on vocabulary, they may have difficulty with mental state terms, emotion words, and deictics (Tager-Flusberg et al., 2005). In reading, although *decoding* (the ability to read words aloud) is (on average) on par with developmental level, reading comprehension is generally impaired (e.g., Nation et al., 2006). Some children with autism have *hyperlexia*, in which decoding outstrips comprehension (Tager-Flusberg et al., 2005).

While some aspects of semantic processing and categorization are intact in autism, others are different or impaired. Unlike controls, autistic children do not use semantic categories to cluster items during recall (e.g., Hermelin and O’Connor, 1970). Children with autism perform similarly to controls on some categorization tasks (e.g., Ungerer and Sigman, 1987); however, unlike the categories of typically developing children, their categories appear to not be based on prototypes (e.g., Dunn et al., 1996). Autistic people (without intellectual disability) showed semantic priming effects in word completion tasks (Toichi and Kamio, 2001) but not on a lexical decision task (Kamio et al., 2007).

Evidence from electrophysiological and neuroimaging studies supports the view that semantic processing is affected in autism. In an ERP study, Dunn et al. (1999) found that children with autism, unlike controls, did not show a N400 response (usually seen in response to semantic incongruity) to unexpected non-target words. In contrast, in a study using magnetoencephalography (MEG), Braeutigam et al. (2008) found that autistic adults without intellectual disability had different MEG patterns for congruent and incongruent sentence endings, though patterns in both conditions were different from those of controls. Neuroimaging studies have found atypical patterns of activation during semantic and other linguistic processing in autism (e.g., Müller et al., 1999; Just et al., 2004; Gaffrey et al., 2007).

#### Role of resource allocation in semantic and pragmatic processing in autism

In terms of the present approach, semantic and pragmatic processing both involve many stages of processing. When resources are limited, earlier stages compete for resources with later, more meaning-related stages. For autistic people with the most reduced or narrowed resources, competition for resources between perceptions and higher levels of processing may lead to difficulty simultaneously allocating resources to the sound and meaning of language; they may have trouble accessing even the literal meanings of words and sentences.

The temporal nature of spoken language, in which the processing of previous words must be completed while new words rapidly come in, may complicate allocating attention to several stages of processing in autism. Similar difficulties may occur in reading. The intact decoding and impaired comprehension characteristic of hyperlexia may reflect a tendency for early stages of processing to use up the available resources.

Because autistic people with increasingly broad attentional bandwidths can attend to progressively more levels of language, the present approach can also help explain the difficulties that people with autism and without intellectual disability are said to have with understanding figurative language, irony, and indirect speech acts. Happé suggests that these difficulties may arise from difficulties in understanding speakers’ communicative intentions; she found that autistic children’s performance on tests of non-literal language was roughly correlated with their performance on first and second-order theory of mind tasks (Happé, 1993, 1995). Gernsbacher and Pripas-Kapit (2012) have criticized these and similar studies, arguing that difficulty in comprehending figurative and contextualized language – and also in doing many theory of mind tasks – may stem from general language comprehension difficulties.

In the present approach, narrowed or reduced resources can lead people with autism to have difficulty in simultaneously attending to literal meanings, figurative meanings, and representations of context. Autistic people’s difficulties with pragmatics may likewise arise from difficulties with simultaneously attending to the literal meanings of utterances, to their social meanings, and to the surrounding social context. In people with Asperger’s disorder, strong interests and linguistic abilities coupled with difficulty in simultaneously talking and tracking interlocutors’ reactions may contribute to a tendency to engage in monologues.

The present approach can also help explain neuroimaging results for semantic processing in autism. In this view, due to narrowed attention or reduced attentional capacity, earlier stages of semantic processing in autism receive atypically more resources, while later stages of processing receive fewer resources; the activation of early stages competes with that of later stages. This is supported by recent neuroimaging studies of semantic and other linguistic processing in autism, which have found increased activation relative to controls in regions associated with earlier processing stages along with decreased activation in regions associated with later stages. For example, Gaffrey et al. (2007) found that during a semantic decision task, men with ASCs and without intellectual disability had increased activation in extrastriate visual cortex and decreased activation in the left inferior frontal gyrus (LIFG) compared with controls. Whereas this was attributed to the use of visual imagery by autistic participants, it could also reflect increased resource allocation to the visual stimuli along with concomitant decreases in resource allocation to later stages of processing.

This is not the only approach to link attentional resources and language processing in autism. Oller and Rascon (1999) propose a detailed semiotic hierarchy and suggest that autistic people at progressively higher levels of functioning can use increasing levels of semiotic resources. Bara et al. (2001) link processing of pragmatics and figurative language with attentional bandwidth; because of the study’s facilitated communication manipulation, some may interpret the results with caution. The *monotropism* approach looks at the narrowing of attention in autism in terms of both perceptual narrowing and the narrowed but strong interests of people with autism (Murray et al., 2005). Connecting monotropism with the present approach, people with autism may be able to allocate more resources to processing streams associated with their interests, allowing deeper processing of those topics.

### SOCIAL COGNITION AND INTERACTION

The hypothesized typical resource allocation could contribute to difficulties with social cognition and interaction in several ways.

#### Theory of mind

Problems with theory of mind, and poor performance on false belief tests, have often been noted in autism (e.g., Baron-Cohen et al., 1985; Baron-Cohen, 1995); there has been much debate about these findings and their implications (e.g., Yirmiya et al., 1998). Atypical resource allocation can indirectly contribute to difficulties with theory of mind by affecting autistic people’s comprehension of language and social situations.

There is evidence that impoverished linguistic experience can affect performance on theory of mind tests: a large percentage of prelingually deaf children raised by parents who are not fluent in sign language (and who thus tend to converse in sign with their children about concrete topics) do poorly on theory of mind tests (e.g., Peterson and Siegal, 1998). If, as discussed above, atypical resource allocation affects autistic children’s comprehension of language and social situations, that would decrease their experience with theory of mind concepts and contribute to difficulty with such concepts and with false belief tests.

#### Joint attention

In typical development, joint attention develops before theory of mind. In autism, certain joint attention behaviors are affected early in development (Mundy et al., 1993); impairments in joint attention and in intersubjectivity more generally (Hobson, 1993) have been suggested as primary deficits in autism. Because young children with autism initiated fewer non-verbal bids than controls to share attention to objects, but initiated a similar number as controls of non-verbal requests for objects, Mundy et al. (1993) argue that autistic children’s difficulties with joint attention are social rather than cognitive. More recently, Mundy and Neal (2001) have hypothesized that deficits in joint attention and social orienting in autism lead to impoverished social input, creating a secondary neural disturbance that may help push the child further off the path of typical development. Mundy et al. (2010) regard joint attention as a process that develops with increasing coordination of information about an object, another’s attention to that object, and one’s own experience of the situation (including interoception and proprioception).

In the present approach, resource limitations could affect the development of joint attention by making it harder to simultaneously attend to another person, an object, and processes within the self such as interoception and proprioception. More generally, by interfering with the awareness of bodily feelings (*somatic markers*), which contribute to emotion, social cognition, and decision-making (Damasio, 1994), attentional narrowing or reduced attentional capacity in autism can contribute to problems with social cognition and executive function. Finally, autistic people’s atypical perceptual experiences (which may partly stem from atypical resource allocation) can affect their ability to experience intersubjectivity with neurotypical people.

#### The mirror neuron system

The mirror neuron system, whose neurons are active when a person or monkey executes an action and when they observe that action, has been proposed as being involved in autism (Williams et al., 2004; Dapretto et al., 2006). In Dapretto et al.’s (2006) fMRI study, when children with autism observed and imitated emotional facial expressions, they had less activity than controls in a number of brain areas, including the pars opercularis, a part of the LIFG previously associated with mirror neuron activity; they had similar activation to controls in facial processing areas such as the fusiform gyrus and greater activation than controls in right visual and left anterior parietal areas. In the present approach, the decreased activation seen in the LIFG, at the apex of the mirror neuron system, as well as in other areas, may result from atypical resource allocation leading to decreased activation of later stages of processing rather than from a specific problem with mirror neurons; it may even be caused by the increased activation of the earlier areas. Decreased connectivity could also be involved. This would not change possible *effects* of decreased activation of the mirror neuron system.

## OTHER POSSIBILITIES AND ALTERNATIVES

Before discussing possible neural bases for the hypothesized differences, some alternatives should be mentioned. The current hypotheses assume that autistic people have the relevant motor or comprehension schemas but cannot access or activate them due to resource problems. But it could be that the autistic people don’t have the schemas, perhaps due to a difficulty in forming prototypes (Klinger and Dawson, 1995, 2001), whether due to atypical resource allocation or from other causes. It’s also possible that people with autism have a specific difficulty with social schemas; this could arise from a number of causes, such as the greater complexity of such schemas, a cascade of effects caused by impaired social orienting (Mundy and Neal, 2001), or an innate inability to form social schemas. Tests of the current hypotheses must take these possibilities into account. While a difficulty in forming schemas might explain some of the symptom areas discussed above, it’s hard to see how it could explain other areas such as sensory hypo- and hypersensitivity, enhanced perceptual discrimination, fragmentary perception, and fluctuating senses.

If, as suggested here, differences in autism narrow or reduce the resources deployed to different stages of processing, this could occur in two ways. First, the earliest perceptual levels could claim the resources first, leaving fewer resources for later stages. Second, either the earlier, more perceptual levels or the higher, more abstract levels could receive attentional resources – just not both at the same time.

## POSSIBLE NEURAL UNDERPINNINGS

There are a number of possible neural bases of the hypothesized atypical resource allocation in autism. While much neurological research on autism (for a review, see Minshew et al., 2005) has focused on specific brain areas, there is an increasing emphasis on factors affecting the whole brain and its systems (e.g., Minshew and Williams, 2007; Müller, 2007).

There has been little work explicitly on the neural bases of resource allocation; some is mentioned below. Because the hypothesized resource is similar to or closely underlies selective attention, the following survey will begin with the dorsoparietal orienting network (e.g., Corbetta and Shulman, 2002; Corbetta et al., 2008) and will move on to other neurological areas and aspects that may affect resource allocation in autism.

### THE NETWORK OF AREAS INVOLVED IN ATTENTIONAL ORIENTING MAY ACT DIFFERENTLY

#### Frontal areas

Superior frontal areas, including the FEF, have been implicated in the attention orienting network (Corbetta and Shulman, 2002; Petersen and Posner, 2012). While Bauman and Kemper’s (1994) postmortem study did not find frontal abnormalities in autism, more recent studies have found atypically narrow minicolumns (Buxhoeveden et al., 2006) and brain overgrowth in the frontal lobes of people with autism (e.g., Herbert et al., 2004). A recent postmortem study found that the brains six of seven autistic children had greater numbers of prefrontal neurons than controls, especially in dorsal prefrontal cortex, beyond what might be expected given the increased brain weights also found in the autistic children (Courchesne et al., 2011).

#### Parietal lobes

As noted above, dorsal parietal areas have also been found to be involved in the top-down deployment of attention (Corbetta and Shulman, 2002; Petersen and Posner, 2012) and are a promising area in autism. One MRI study found that 43% of a sample of autistic people had parietal abnormalities (Courchesne et al., 1993); as discussed above, some autistic people with parietal abnormalities as well as cerebellar abnormalities appear to have narrowed visual attention (Townsend and Courchesne, 1994).

In addition, damage to parietal cortex, especially to right parietal cortex, can lead to extinction, a form of which has been suggested as a cause for autistic people’s atypical sensory and attentional experiences (Bonneh et al., 2008). In their case study, Bonneh et al. hypothesize that many of their participant’s extinction-like symptoms and unusual sensory experiences come from a winner-take-all mode of processing in which a salient stimulus or representation extinguishes other stimuli, in what could be seen as an extreme version of the processes described in the biased competition approach (Desimone and Duncan, 1995). They suggest that this pattern as well as slowed attentional shifting may stem from abnormalities in the parietal cortex or superior temporal sulcus.

Could these extinction-like phenomena stem from narrowed attention or reduced attentional capacity? Bonneh et al. (2008) argue that “mono-channel perception” is unlikely to come from a lack of attentional resources because it was found even when perceptual load and attentional demands appeared to be low; they acknowledge that the perceptual load in autism may be higher than it seems. Another question is whether winner-take-all processing or extinction could occur in relation to competition between *different stages of processing* of a single stimulus. This would suggest another possible mechanism for the phenomena highlighted in the present approach.

#### The cerebellum

While some suggest that the cerebellum fine-tunes attentional shifts in the same way that it fine-tunes motor movements and describe morphological changes in the cerebellum in autism (Courchesne, 1989; Courchesne et al., 1994), others have said that the morphological results were correlated with IQ and have not been replicated as specific to autism (Minshew et al., 2005). In addition, the role of the cerebellum in attention has been disputed (e.g., Haarmeier and Thier, 2007); further study is needed.

### OTHER NEURAL AREAS, SYSTEMS, AND PHENOMENA THAT MAY AFFECT RESOURCE ALLOCATION

#### The nucleus reticularis of the thalamus

Though not frequently discussed in relation to attention, the nucleus reticularis of the thalamus (NRT) could play a role in atypical resource allocation in autism. Almost all sensory input passes through the thalamus on its way to the cortex; information may go back through the thalamus several times after processing in various cortical areas. Scheibel (1997) has suggested that the NRT, a thin layer of (inhibitory) GABAergic cells around several sides of the thalamus, is involved in the top-down control of pain and the gating of sensory input to the cortex. NRT cells are part of a complex feedback system involving prefrontal cortex, thalamic nuclei, and the midbrain tegmentum.

There are several ways that the NRT might be involved in sensory and attentional phenomena in autism. For sensory information coming back from the cortex to the thalamus, the presence of numerous narrow cortical minicolumns (Casanova et al., 2002) could lead to excessive input to areas of the NRT, leading to atypical inhibition of surrounding areas and possibly to narrowed attention. Conversely, deficits in GABAergic neurons hypothesized in autism (Rubenstein and Merzenich, 2003), or decreased input from the midbrain tegmentum, might mean less NRT activity, flooding the cortex with input; this could correspond to the sensory overload sometimes reported in autism. Atypical prefrontal input to the NRT could also affect sensory processing in autism.

#### Limbic system

Due to their role in emotion and cognition, limbic areas have long been suspected to be involved in autism. Bauman and Kemper (1994) found abnormalities in most limbic areas in autism, including impoverished dendritic arbors in hippocampal pyramidal cells. Waterhouse et al. (1996) suggest that these hippocampal abnormalities may result in *canalesthesia*, in which cross-modal processing of events and memories is fragmented; they suggest that the hippocampus may indirectly affect attention in autistic people through its feedback to cortical areas.

#### Laterality

Hemispheric specialization and interhemispheric communication, both of which appear to be affected in ASCs, are closely related to issues of resource allocation. Hemispheric specialization for a variety of functions is thought to increase processing efficiency, minimizing resource use. According to Friedman and Polson (1981), evidence generally supports the view of hemispheres as independent pools of resources. In addition, the optimal division of labor between the hemispheres varies depending on task conditions (Zaidel et al., 1988) and has been shown to change after sleep deprivation, when efficiency and resources are presumably reduced (Coto, 2009). In terms of the present approach, altered laterality is most relevant to the hypothesis of reduced attentional capacity.

Behavioral studies of hemispheric specialization in autism have had mixed results (e.g., Prior and Bradshaw, 1979; Dawson et al., 1986; Rumsey and Hamburger, 1988). Rinehart et al. (2002) argue that the autistic profile has elements of both left hemisphere dysfunction (impaired language and sequential processing; preserved visual-spatial and musical abilities) and right-hemisphere dysfunction (impaired pragmatics and prosody; relatively preserved syntax and phonology). People with autism (without intellectual disability), especially those with early language problems, have less clearly established handedness than controls (Escalante-Mead et al., 2003).

Neurological evidence is likewise mixed, including evidence about whether the corpus callosum is smaller in autism (Minshew et al., 2005); there is some evidence of reduced inter-hemispheric information transfer (Nydén et al., 2004). Structural MRI has found atypical brain asymmetry in autistic boys with language impairment, while those without language impairment were similar to controls (De Fossé et al., 2004). Different patterns of hemispheric activation found in ASCs and controls during language processing depend on the task and do not fall into a simple hemispheric pattern (e.g., Müller et al., 1999; Just et al., 2004; Harris et al., 2006; Kleinhans et al., 2008). The general picture regarding laterality for language in ASCs is one of decreased hemispheric specialization and increased right-hemisphere involvement relative to controls, especially for autistic people with language impairments.

Atypical laterality in ASCs could be involved in atypical resource allocation in several ways. Decreased hemispheric specialization could lead processing to be less efficient, “using up” more resources. Conversely, inefficient processing might lead people with autism to use both hemispheres for tasks only requiring one hemisphere in neurotypical people. Even if hemispheric specialization for certain functions is fairly typical in ASCs, reduced resources or decreased interhemispheric connectivity might largely confine the receptive processing of a stimulus to one hemisphere, to the detriment of processes associated with the other hemisphere or requiring hemispheric cooperation. These possibilities can be tested using experiments with unilateral and bilateral visual hemifield presentations as well as with ERPs and neuroimaging (e.g., Zaidel et al., 1988; Narr et al., 2003; Coto, 2009).

### INTENSE SENSORY PROCESSING MAY AFFECT OTHER STAGES OF PROCESSING

The atypical sensory experiences and enhanced perceptual discrimination discussed earlier imply that sensory processing is sometimes more intense and detailed in autism than in typical development. As noted in Samson et al.’s (2012) ALE study, some neuroimaging studies of higher-level processing in autism have found more activation in early perceptual areas in ASC participants than in controls (e.g., Just et al., 2004; Koshino et al., 2005). Intense sensory processing in autism could be related to several other neural phenomena: people with autism have been found to have narrower, more numerous cortical mini-columns (Casanova et al., 2002), and have been hypothesized to have a greater ratio of neural excitation to inhibition (Rubenstein and Merzenich, 2003), increased local connectivity (e.g., Belmonte and Yurgelun-Todd, 2003), and hyperactive local circuits (Markram et al., 2007).

Increased processing in a primary sensory area could lead to decreased processing in other sensory areas and at higher levels due to resource-allocating mechanisms. Though little is known about such mechanisms, several lines of research imply that they exist. First, behavioral experiments by Lavie (1995) indicate that under conditions of high perceptual load, selective attention occurs earlier in the system and more irrelevant items are screened out; thus, intense sensory processing in autism could lead to narrowed attention^10^.

Second, as mentioned earlier, fMRI experiments have found that attention to one feature or modality can reduce the activation of areas processing other features or modalities (e.g., Corbetta et al., 1990; Shomstein and Yantis, 2004). Third, in a phenomenon known as “negative BOLD,” the activation of one area of visual cortex can lead to decreased activation of other areas; this decreased activation appears to result from a neural control mechanism rather than from the mechanical “stealing” of blood flow by the activated area (Smith et al., 2004). The hemodynamic response itself, in which neural activation leads to increased blood flow to a brain area, might be different in autism. Compared with mental-age-matched controls, children with both intellectual disability and autism have been found to have reduced perfusion in a number of brain areas (e.g., Ohnishi et al., 2000; Zilbovicius et al., 2000); however, the participants were sedated and the findings not consistent. In any event, it is possible that atypically intense sensory activation coupled with typical mechanisms of resource allocation could lead to decreased activation of other sensory modalities and of later stages of processing.

### ATYPICAL BRAIN CONNECTIVITY MAY BE INVOLVED

An increasing number of studies have suggested that atypical brain connectivity is involved in autism. Based on studies of functional connectivity, a number of researchers have suggested that there is *underconnectivity* in autism – decreased long-range connectivity between brain areas. For example, in their fMRI study of sentence processing, Just et al. (2004) found that compared with controls, autistic participants had decreased functional connectivity between a variety of pairs of brain areas, most including frontal areas. As noted by Müller (2007), however, not all functional connectivity studies of autism have found evidence for general underconnectivity.

Citing work on structural as well as functional connectivity, other studies have suggested that in addition to long-range underconnectivity, there is local *overconnectivity* in autism (Belmonte and Yurgelun-Todd, 2003; Courchesne and Pierce, 2005). Belmonte and Yurgelun-Todd suggest that such increased local connectivity in autism is associated with intense perceptual activation, impaired selective attention, and a poor signal-to-noise ratio, leading to inefficient compensation at higher levels of processing. Courchesne and Pierce (2005) argue that findings in autism of increased white matter, inflammation, and atypical minicolumn patterns in frontal areas imply that excessive connectivity within the frontal lobes may be coupled with reduced connectivity to more posterior regions. Similarly, Geschwind and Levitt (2007) suggest that atypical cell growth early in autism may lead evolutionarily recent higher association areas to be disconnected from evolutionarily older sensory areas. Finally, Markram et al. (2007) suggest that increased connectivity, reactivity, and plasticity of neocortical microcircuits in autism could lead to intense perception, attention, and memory as well as decreased frontal coordination.

In terms of the present approach, increased local structural connectivity could lead to intense sensory processing, which could affect later stages of processing as discussed above. Decreased long-distance structural connectivity could cause sensory signals to become atypically attenuated as they move to later stages of processing; it could also impair feedback to earlier areas, as suggested by Frith (2003). Nevertheless, the present approach differs from purely connectivity-based approaches. While many connectivity approaches emphasize structural as well as functional connectivity, the attentional differences hypothesized in the present approach (though they may be partly caused by atypical structural connectivity) rely more on the fluid deployment of attentional resources. This may better explain why individuals with autism have different experiences and abilities at different times. The present approach accounts for the processing of meaningful stimuli in terms of the simultaneous activation of different stages of processing, while Just et al.’s (2004) approach emphasizes the coordination of higher-level brain areas; the two accounts are not mutually exclusive.

## TESTING THE HYPOTHESES

In this section, I will sketch several experimental approaches that can help test and refine the hypotheses. In addition to seeing whether the hypotheses hold for autistic people generally, it will be useful to test subgroups of autistic people in which atypical resource allocation is likely to be a greater factor, such as those with strong sensory symptoms (hyposensitivity and/or hypersensitivity), and to examine correlations between each finding and measures of sensory symptoms.

### USE RESOURCE THEORETICAL TECHNIQUES TO EXAMINE WHETHER RESOURCES IN AUTISM MARE NARROWED OR REDUCED

A preliminary question is whether the structure of resources in autism is similar to the structure found in typical development, as exemplified in Wickens’s 3-D + 1 model (Wickens, 2002, 2008).

The structure of resources in autism can be tested using techniques similar to those used in typical development (e.g., Navon and Gopher, 1979; Wickens, 1980, 1984, 2002, 2008). Because tasks can be resource-limited (limited by the amount of resources) up to the application of a certain amount of resources and data-limited (limited by the quality of the data) thereafter (Norman and Bobrow, 1975), tasks are best tested at different levels of resources. This is generally done using dual-task paradigms (e.g., Navon and Gopher, 1979; Wickens, 1980, 1984) in which the priority or the difficulty (or both) of a manipulated task is varied and its effects on performance on a measured task are examined. Performance on each task done singly is also examined, providing a limiting case in which no resources are taken by the other task. By varying the type of task (e.g., auditory vs. visual; input- or output-focused) and seeing which kinds of tasks interfere with each other, the structure of resource pools can be inferred.

Dual-task experiments face a number of potential issues, including the possibility that results may reflect interference between tasks (Navon, 1984) or the cost of switching back and forth between the tasks rather than the simultaneous use of resources. These challenges are exacerbated when studying resource allocation in affected populations; analogous issues arise when dual-task experiments are used to examine resources across development (Guttentag, 1989). Some concerns are that members of the two groups may implement priority instructions differently (e.g., may not be able to allocate 30% of their attention to one task and 70% to another) and may have different abilities (e.g., autistic participants may be better than neurotypical controls at some perceptual tasks and worse at some verbal ones). To allocate different amounts of resources to the tasks, participants have to be able to understand the instructions, and should probably be adolescents or adults (Irwin-Chase and Burns, 2000, found that in a dual-task paradigm, children before the fifth grade could not make more subtle attention allocations than 50–50).

One of the few dual-task studies of autism illustrates both the promise and possible problems of these paradigms. In a study arising out of work on the executive functions of working memory, Garcia-Villamisar and Della Sala (2002) found that when adults with autism did a digit recall task along with a tracking task (in which they crossed out boxes arranged in paths on pieces of paper), their performance on both tasks declined relative to their performance on either task alone, while controls were not so affected. The findings support the present hypotheses of reduced resources. Note that the two tasks use fairly separate resource pools in Wickens’s 3-D+1 scheme: the digit recall task involves the auditory modality, perceptual/cognitive resources (memory), and vocal responses; the tracking task involves the visual modality and manual responses.

However, the results for the autistic participants may also reflect problems with executive function, such as difficulties in organizing themselves to do both tasks or in shifting between tasks; autistic participants have difficulty in shifting between tasks and modalities (e.g., Reed and McCarthy, 2012). In all participants, a measure of combined performance was negatively correlated with a questionnaire measuring executive function (*r* = -0.323), suggesting that executive function may indeed play a role. A weaker correlation between combined performance on the tasks and the Wisconsin Card Sort Test (*r* = -0.16) suggests that perseveration or shifting set was not the main cause of the dual-task deficits. Varying the priority or difficulty of one task would also help determine whether the reduced performance on both tasks in autism reflects one task taking resources from the other or the “concurrence cost” of doing any two tasks at once.

Despite the above concerns, dual-task experiments can help compare the structure and allocation of resources in autism and in typical development. Such experiments can systematically focus on dimensions in Wickens’s (2002, 2008) 3-D + 1 schema that are of particular interest in the present approach, such as whether in the general perceptual pool, different modalities such as vision and hearing appear to be in separate pools in autism as they are in typical development, or likewise, whether the perception/cognition pool is separate from the response pool.

### USE OTHER BEHAVIORAL PARADIGMS TO EXAMINE RESOURCE ALLOCATION IN AUTISM

#### Reducing resource demands in tasks with multiple levels of processing

If narrowed attention or reduced attentional capacity leads to difficulty in autism in simultaneously allocating resources to different stages of processing, then for tasks with multiple stages of processing, manipulations that free up resources should improve processing at later stages. For instance, making earlier stages of linguistic processing less attention-grabbing (for example, by presenting spoken stimuli more quietly or in a monotone) might improve semantic and pragmatic processing in people with autism more than in controls. One would have to take into account participants’ sensory discrimination abilities, their physiological and emotional responses to stimuli, and the amount of information contained in the stimuli.

#### Trade-offs between levels of processing

The hypotheses of narrowed or reduced attention imply that for each (sufficiently difficult) stimulus, people with autism will have a tradeoff between different stages of processing, while controls will not. One can test this prediction by following stimuli with probes that measure processing at different stages, and examining correlations between measures of performance associated with the different stages. While neurotypical participants would tend to have positive correlations between measures of performance at different stages (because they would either attend to each stimulus or not), people with autism might have negative correlations (because their attention to one stage of processing would compete with their attention to other stages). For example, one could present a series of words known to all participants, and after each word present probes examining acoustic, phonological, and semantic processing. The variable of interest would not be the participants’ overall performance on the each kind of probe, but rather, correlations between performance on different probes for each stimulus for each participant.

### USE NEUROIMAGING TO TEST THE HYPOTHESES

Neuroimaging techniques present promising ways to test the hypotheses and explore both processing trade-offs. According to the hypothesis that narrowed attention can affect resource deployment to different stages of processing, when compared with neurotypical people, people with ASCs should have increased activation of early processing areas, including those associated with the input modalities of symbolic stimuli, coupled with decreased activation of later processing areas. Thus, for visually presented linguistic stimuli, one would expect greater activation in early visual areas in ASC participants relative to controls, while for auditorially presented linguistic stimuli, one would expect greater activation in early auditory processing areas (Two caveats: if, as suggested by Damasio, 1994, words and concepts have meaning by reactivating early sensory processing areas associated with their referents, one would have to distinguish between the activation of sensory areas by sensory input and their reactivation by higher-level schemas. Also, more generally, each technique has an indirect relationship to neural activity, and such activity may not always reflect attention or resource deployment).

Existing neuroimaging work, much of it summarized in Samson et al.’s (2012) meta-analysis, provides some support for these predictions. For example, Just et al.’s (2004) finding of increased activation in Wernicke’s area coupled with decreased activation in Broca’s area during sentence processing in autistic participants without intellectual disability may reflect a resource tradeoff. Gaffrey et al.’s (2007) finding of increased activation in the early visual areas of participants with ASCs during the processing of visually presented linguistic stimuli may reflect increased activation of the input modality.

At shorter time-scales, the hypotheses also predict negative correlations between the activation of early and late processing areas in people with ASCs but not in controls. It is unclear, however, whether resource allocation mechanisms act at short enough time scales that *functional connectivity* techniques would find such negative correlations; to my knowledge, no such results have been reported.

### FALSIFYING THE HYPOTHESES

Because of the difficulties of testing resource theories and the many possible causes of the hypothesized atypical resource allocation, it would be hard to disprove the hypotheses with a single, disconfirmatory experiment. Nonetheless, convergent disconfirmatory evidence would disprove the hypotheses, especially if it was found in autistic people with strong sensory symptoms, who the theory predicts are most likely to have atypical resource allocation as a factor.

Two findings that would weigh against the hypothesis would be similar performance by autistic people and controls on a variety of dual-task experiments using different modalities and levels of difficulty, and an absence of negative correlations in autistic people among measures of performance at different levels of processing of difficult meaningful stimuli. In addition, findings supporting convincing alternative explanations of phenomena focused on in this account (e.g., sensory hyper- and hyposensitivity, difficulties with comprehension, and difficulties with action) would weaken the hypotheses for those areas.

In terms of neuroimaging, assuming that the functions of brain areas in autism are roughly similar to their functions in typical development, a finding that activation associated with earlier stages is not increased or spared and that activation associated with later stages of processing is not reduced would weigh against the hypotheses.

## CONCLUSION

I have presented two hypotheses about how atypical resource allocation in ASCs could affect perceptual processing, the processing of meaningful stimuli, and the control of action. I have hypothesized that, especially in autistic people with strong sensory symptoms, attentional narrowing or reduced attentional capacity lead to atypical resource allocation both within perception and to different stages of processing of stimuli. I have suggested that this atypical resource allocation contributes to autistic people’s difficulties with language and social cognition, to their issues with executive function and movement, to their atypical sensory and attentional experiences, and to their perceptual/conceptual strengths and weaknesses. Possible neural bases for atypical resource allocation include differences in the systems that control attention, the cascading effects of intense sensory processing, and atypical connectivity. Ways to test and refine the hypotheses include dual-task experiments and the use of experimentation and neuroimaging to determine whether people with autism have negative correlations between measures of different stages of processing.

The approach has a number of limitations; a few will be mentioned. First, the nature of the resources is left open; there are many possible instantiations, and thus more possibilities to test. Second, it’s hard to test resource theories; other phenomena such as executive difficulties or cross-talk (interference) between processes could lead to similar results. Thus, though the suggested experiments would provide evidence for or against the hypotheses, none of them is definitive, and convergent evidence is needed. Some phenomena discussed here, such as aspects of EPF, may be caused by structural differences such as altered lateral connectivity in early processing areas (Kéïta et al., 2011). Nevertheless, such wiring differences may affect resource allocation at later stages, helping explain more changeable aspects of autism such as the experience of sensory fluctuation. Finally, atypical resource allocation is hypothesized to be only one factor in autism; many factors are likely to contribute to the heterogeneity in ASCs.

Several other approaches to autism focus on strengths and weaknesses in autism in a somewhat similar way. These include approaches centered on complexity (e.g., Minshew and Goldstein, 1998), connectivity (e.g., Just et al., 2004), competition (Bonneh et al., 2008), hyper-processing (Markram et al., 2007), and EPF (e.g., Bertone et al., 2005; Mottron et al., 2006). Most of these approaches are not mutually exclusive. For instance, it’s possible that in autism, both reduced structural or functional connectivity and atypical resource allocation lead to a reduction in the activation of higher-level processing areas. It’s also possible that different subgroups of people with ASCs have different etiologies, but that the increased activation of lower-level processing areas coupled with diminished activation of higher-level processing areas is a final common pathway.

If the atypical resource allocation is found to be a factor in many aspects of autism, we may be better able to understand the causes and consequences of atypical sensory and attentional experiences common in the syndrome, and to help people with autism allocate their resources differently when they wish to. We may be able to predict what makes stimuli easy or hard to process in autism, and to use this information in designing educational programs for people with autism. Finally, we may be better able to understand autistic people’s strengths as well as their weaknesses.

## Conflict of Interest Statement

The author declares that the research was conducted in the absence of any commercial or financial relationships that could be construed as a potential conflict of interest.

## Footnotes

1 An earlier version of this approach is detailed in Goldknopf (2006).

2 Sensory symptoms are now part of the DSM-V criteria for autism (American Psychiatric Association, 2013).

3 A related hypothesis, “difficulty with subordination to a schema” (Goldknopf, 2006), will not be discussed here due to space constraints. That hypothesis states that in typical cognition, resources for perceptions and lower-level schemas are decreased in favor of resources for higher-level schemas; in autism, resources for perceptions and lower stages may not be decreased in favor of resources for higher ones.

4 This simplified picture omits prosody, which is largely processed by the right hemisphere (Bookheimer, 2002).

5 An intriguing recent approach links cholinergic systems to “attentional effort” and to performance on attentional tasks (Sarter et al., 2006).

6 Another approach to selective attention, the biased competition approach, emphasizes bottom-up processes but also allows for frontal and parietal biasing (Desimone and Duncan, 1995; Pessoa et al., 2003).

7 Though joint attention will be discussed later, the extensive work on socially cued attention in autism is beyond the scope of this paper. Two recent reviews include discussion of this area (Simmons et al., 2009; Ames and Fletcher-Watson, 2010).

8 The results may also have reflected difficulties in shifting set.

9 More recently, Bonnel et al. (2010) found enhanced discrimination of simple auditory stimuli in autism without decreased discrimination of complex stimuli.

10 This last suggestion is not supported by Remington et al. (2009), who found that adults with autism had greater perceptual capacity than controls. (Controls, however, had less perceptual capacity than neurotypical people in other studies).
